# A modified Ranson score to predict disease severity, organ failure, pancreatic necrosis, and pancreatic infection in patients with acute pancreatitis

**DOI:** 10.3389/fmed.2023.1145471

**Published:** 2023-06-02

**Authors:** Xiuping Luo, Jie Wang, Qing Wu, Peng Peng, Guolin Liao, Chenghai Liang, Huiying Yang, Jiean Huang, Mengbin Qin

**Affiliations:** ^1^Department of Gastroenterology, The Second Affiliated Hospital of Guangxi Medical University, Nanning, China; ^2^Department of Gastroenterology, The First Affiliated Hospital of Guangxi Medical University, Nanning, China

**Keywords:** acute pancreatitis, modified Ranson score, Ranson score, bedside index of severity in acute pancreatitis, modified computed tomography severity index

## Abstract

**Background:**

Although there are several scoring systems currently used to predict the severity of acute pancreatitis, each of them has limitations. Determine the accuracy of a modified Ranson score in predicting disease severity and prognosis in patients with acute pancreatitis (AP).

**Methods:**

AP patients admitted or transferred to our institution were allocated to a modeling group (*n =* 304) or a validation group (*n =* 192). A modified Ranson score was determined by excluding the fluid sequestration parameter and including the modified computed tomography severity index (CTSI). The diagnostic performance of the modified Ranson score was compared with the Ranson score, modified CTSI, and bedside index of severity in acute pancreatitis (BISAP) score in predicting disease severity, organ failure, pancreatic necrosis and pancreatic infection.

**Results:**

The modified Ranson score had significantly better accuracy that the Ranson score in predicting all four outcome measures in the modeling group and in the validation group (all *p* < 0.05). For the modeling group the modified Ranson score had the best accuracy for predicting disease severity and organ failure, and second-best accuracy for predicting pancreatic necrosis and pancreatic infection. For the verification group, it had the best accuracy for predicting organ failure, second-best accuracy for predicting disease severity and pancreatic necrosis, and third-best accuracy for predicting pancreatic infection.

**Conclusion:**

The modified Ranson score provided better accuracy than the Ranson score in predicting disease severity, organ failure, pancreatic necrosis and pancreatic infection. Relative to the other scoring systems, the modified Ranson system was superior in predicting organ failure.

## Background

Acute pancreatitis (AP) is a common inflammatory disorder of the pancreas that has numerous causes, and is characterized by different degrees of involvement of other regional tissues or remote organ systems. The global incidence of AP is about 34/100,000, and its incidence has increased over time (1). According to the AP Grading and Classification System from the 2012 International AP Symposium in Atlanta, AP can be classified by clinical manifestations and prognosis as mild AP, moderate-severe AP, or severe AP (SAP) (2). The fatality rate from SAP ranges from 36% to 50% (3, 4). Therefore, to improve the care of these patients, it is important to have a tool that can stratify patients according to their risk of progression to SAP.

There are several scoring systems currently used to predict the severity of AP, including as bedside index of severity in acute pancreatitis (BISAP) score, the Ranson score, the acute physiology and chronic health evaluation II (APACHE-II) score, and the modified computed tomography severity index (CTSI). However, each of these scoring systems has limitations. For example, two systematic reviews reported lower specificity of the APACHE II score than the Ranson score in predicting SAP (5, 6). Secondly, the APACHE II scoring system has more variables than the Ranson score, making its application more difficult. Compared with the Ranson score, the BISAP score has fewer variables and is easier to calculate. However, the BISAP requires assessment of mental status, and this is often subjective and requires comparison with mental status at baseline, making its use problematic for patients with a long history of disease. The modified CTSI score is also limited in that it considers peripancreatic necrosis and pseudocyst formation, most of which are not present during the early stages of AP; in addition, the CTSI score must be conducted 2 to 3 days after admission, thus slowing the diagnosis and potentially delaying early treatment (7). Ong and Shelat showed that the Ranson score had accuracy that was comparable or superior to that of other commonly used scoring systems (APACHE II, BISAP and CTSI) in stratifying the severity of AP and predicting mortality, based on a systematic review (8).

Researchers first developed the Ranson score from analysis of 43 clinical and laboratory parameters that were recorded within 48 h of hospital admission in 100 patients who had AP (9–11). They ultimately determined the final score from five parameters recorded at admission (age, white cell count, blood glucose, aspartate transaminase, and lactate dehydrogenase) and six parameters recorded at 48 h after admission (reduction in hematocrit, increase in blood urea nitrogen, serum calcium, arterial PO_2_, base deficit, and fluid sequestration) (9, 12) However, many studies concluded that calculation of the Ranson score is time-consuming, has low predictive value (13), and has little practical clinical value. Others have criticized use of the Ranson score because it takes 48 h to calculate and may therefore lead to treatment delays. However, for a patient whose AP severity is not evident within 48 h after admission, variables recorded at 48 h may help in reassessment, making it possible to follow disease dynamics and more reliably predict the persistence and severity of multiple organ dysfunction. Therefore, a new, simple, and accurate clinical scoring system is needed to evaluate and predict the severity of AP.

Our general purpose was to develop a new tool that can be used to guide early clinical interventions and reduce the mortality from AP. Thus, we first developed a new scoring system—the modified Ranson score—that is derived from the original Ranson score. We then analyzed and compared the diagnostic value of the new Ranson score with the original Ranson score, the BISAP score, and the modified CTSI in predicting the severity and prognosis of AP.

## Methods

### Definitions

All patients were classified as having mild or severe AP according to the 2012 AP Grading and Classification System, and based on the presence of organ failure for 48 h or more and/or local complications (2).

### Ethics statement

This study was approved by the Ethical Review Committee of the Second Affiliated Hospital of Guangxi Medical University [Approval Number: 2020-KY (0136)].

### Research subjects

Consecutive AP patients who were hospitalized in the Second Affiliated Hospital of Guangxi Medical University from June 2017 and October 2021 were included. At the time of discharge or death, patients were graded as having mild AP, moderate-severe AP, or SAP. All included patients were more than 18 years-old and had complete clinical data. Patients were excluded if they had features of acute-on-chronic AP based on history or imaging; onset of AP more than 72 h after admission; duration of hospital stay less than 48 h; or unavailability of any data required for calculating AP severity scores because of referral from another institution.

### Assignment of diagnostic scores

Extensive demographic, radiographic, and laboratory data were recorded from patients who were admitted or transferred to our institution because of AP. The modeling group consisted of 304 patients who were hospitalized from June 2017 to March 2020, and the validation group consisted of 192 patients who were hospitalized from April 2020 to October 2021. The original Ranson score has a maximum of 11, and considers the subjective index of fluid sequestration at 48 h. The modified CTSI score (maximum of 10) was determined within 48 h of admission, and patients were assigned scores of 0 (0 points), 1 (1–3 points), 2 (4–6 points), or 3 (7–10 points). The modified Ranson score (maximum of 13) was calculated by excluding the fluid sequestration parameter and including the modified CTSI score. The optimal cut-offs for each scale were determined from the ROC analysis and calculation of the Youden index. According to this cut-off, patients were divided into two groups: low-grade modified Ranson score (modified Ranson score <3) and high-grade modified Ranson score (modified Ranson score ≥3). These two groups were compared in terms of baseline data and clinical outcomes. In addition, the AUC, specificity, and sensitivity of the modified Ranson score, Ranson score, BISAP, and modified CTSI for predicting disease severity, organ failure, pancreatic inflammation, and pancreatic necrosis were calculated in the modeling and verification groups.

### Statistics

Data were recorded in a Microsoft Excel spreadsheet. Variables with non-normal distributions were expressed as medians and inter-quartile ranges (IQRs) and were compared using the Mann–Whitney *U* rank-sum test. Variables with normal distributions were compared using the *χ*^2^ test or Fisher’s exact test. Receiver operating characteristics (ROC) curves were drawn using MedCalc version 19.0. ROC curves for SAP and organ failure were plotted for the modified Ranson score, Ranson score, BISAP, and modified CTSI, and the predictive accuracy of each score was expressed as area under ROC curve (AUC) with 95% confidence interval. Pairwise comparisons of AUC values were performed using the *Z*-test. A *p*-value below 0.05 was considered significant.

## Results

### Patient characteristics

We enrolled 496 patients who had AP, 220 with low-grade AP (158 males and 62 females) and 276 (208 males and 68 females) with high-grade AP based on the modified Ranson score (Table 1). These two groups had no significant differences in gender, smoking, hypertension, or diabetes. However, the high-grade group was older (*z* = −3.992, *p* < 0.05); had different etiologies (*χ*^2^ = 15.59, *p* < 0.05), longer hospitalization (*z* = −5.765, *p* < 0.001), and greater hospitalization costs (*z* = −7,230, *p* < 0.001); and had higher prevalences of drinking alcohol (*χ*^2^ = 5.801, *p* < 0.05), pancreatic infection (*χ*^2^ = 91.476, *p* < 0.001), pancreatic necrosis (*χ*^2^ = 27.980, *p* < 0.001), organ failure (*χ*^2^ = 9.971, *p* = 0.0016), and severe AP (*χ*^2^ = 131.792, *p* < 0.001).

**Table 1:** Characteristics of patients who had high-grade or low-grade acute pancreatitis (*n =* 496).^a^

Characteristic	High-grade (*n =* 276)	Low-grade (*n =* 220)	Statistic	*p*-value
**Gender**			*χ*^2^ = 0.793	0.3731
Male	208 (75.36%)	158 (71.82%)		
Female	68 (24.64%)	62 (28.18%)		
**Age**	48.5 (36.0, 64.0)	41.5 (32.0, 54.0)	*z* = −3.992	0.0001
**Smoking**	144 (52.17%)	106 (48.18%)	*χ*^2^ = 0.779	0.3775
**Drinking**	158 (57.25%)	102 (46.36%)	*χ*^2^ = 5.801	0.0160
**Diabetes**	53 (19.20%)	38 (17.27%)	*χ*^2^ = 0.304	0.5815
**Hypertension**	25 (9.06%)	10 (4.55%)	*χ*^2^ = 3.793	0.0515
**Fatty liver**	99 (35.87%)	55 (25.00%)	*χ*^2^ = 6.886	0.0087
**Etiology**			*χ*^2^ = 15.590	0.0014
Gallstones	115 (41.67%)	93 (42.27%)		
Hypertriglyceridemia	85 (30.80%)	42 (19.09%)		
Alcoholic	36 (13.04%)	33 (15.00%)		
Unclear	40 (14.49%)	52 (23.64%)		
**Hospitalization, days**	9 (6, 13)	7 (5, 10)	*z* = −5.765	0.0001
**Hospitalization cost, RMB**	20866.290 (11548.680, 32788.460)	11043.905 (7146.175, 20683.265)	*z* = −7.230	0.0001
**Pancreatic infection**	252 (91.30%)	118 (53.64%)	*χ*^2^ = 91.476	0.0001
**Pancreatic necrosis**	49 (17.75%)	6 (2.73%)	*χ*^2^ = 27.980	0.0001
**Organ failure**	58 (21.04%)	23 (10.45%)	*χ*^2^ = 9.971	0.0016
**Disease severity**			*χ*^2^ = 131.792	0.0001
MAP	106	196		
MSAP+SAP	170	24		

MSAP, moderately severe acute pancreatitis; SAP, severe acute pancreatitis.

a Values are given as *n* (%) or median (IQR).

### Characteristics of the modeling and verification groups

The modeling group consisted of 304 patients who were hospitalized from June 2017 to March 2020 and the validation group consisted of 192 patients who were hospitalized from April 2020 to October 2021 (Table 2). These two groups had no significant differences in age, use of alcohol, diabetes, hypertension, fatty liver, etiology, pancreatic necrosis, organ failure, or severity of AP. However, the modeling group had more females (*χ*^2^ = 4.672, *p* = 0.0306), longer hospitalization (*z* = −3.872, *p* = 0.001), higher hospitalization costs (*z* = −2.573, *p* = 0.0101), and higher prevalences of smoking (*χ*^2^ = 12.540, *p* = 0.0004) and pancreatic infection (*χ^2^* = 19.354, *p* < 0.0001).

**Table 2:** Characteristics of the modeling group and verification group.^a^

Characteristic	Modeling group (*n =* 304)	Validation group (*n =* 192)	Statistic	*p*-value
**Sex**			*χ*^2^ = 4.672	0.0306
Male	214 (70.39%)	152 (79.17%)		
Female	90 (29.61%)	40 (20.83%)		
**Age**	47.4 (45.6, 49.3)	46.1 (43.7, 48.5)	*z* = −0.873	0.3829
**Smoking**	134 (44.08%)	116 (60.42%)	*χ*^2^ = 12.540	0.0004
**Drinking**	157 (51.65%)	103 (53.65%)	*χ*^2^ = 0.189	0.6640
**Diabetes**	50 (16.45%)	41 (21.35%)	*χ*^2^ = 1.891	0.1690
**Hypertension**	20 (6.58%)	15 (7.81%)	*χ*^2^ = 0.487	0.4850
**Fatty liver**	99 (32.57%)	55 (28.65%)	*χ*^2^ = 0.845	0.3580
**Etiology**			*χ*^2^ = 6.810	0.0780
Gallstones	131 (43.09%)	77 (40.11%)		
Hypertriglyceridemia	67 (22.04%)	60 (31.25%)		
Alcoholic	42 (13.82%)	27 (14.06%)		
Unclear	64 (21.05%)	28 (14.58%)		
**Hospitalization, days**	9 (6–12)	7 (5–11)	*z* = −3.872	0.0001
**Hospitalization costs, RMB**	16688.7500 (10054.205, 29610.775)	12358.0450 (8221.915, 25338.820)	*z* = −2.573	0.0101
**Pancreatic infection**	204 (67.1%)	166 (86.46%)	*χ*^2^ = 19.354	<0.0001
**Pancreatic necrosis**	29 (9.54%)	26 (13.54%)	*χ*^2^ = 1.912	0.1670
**Organ failure**	46 (15.13%)	35 (18.23%)	*χ*^2^ = 0.826	0.3630
**Disease severity**			*χ*^2^ = 0.001	0.9854
MAP	185	117		
MSAP+SAP	119	75		

MSAP, moderately severe acute pancreatitis; SAP, severe acute pancreatitis.

a Values are given as *n* (%) or median (IQR).

### Roc analysis of the modeling group using the four scoring systems

**Figure 1:**
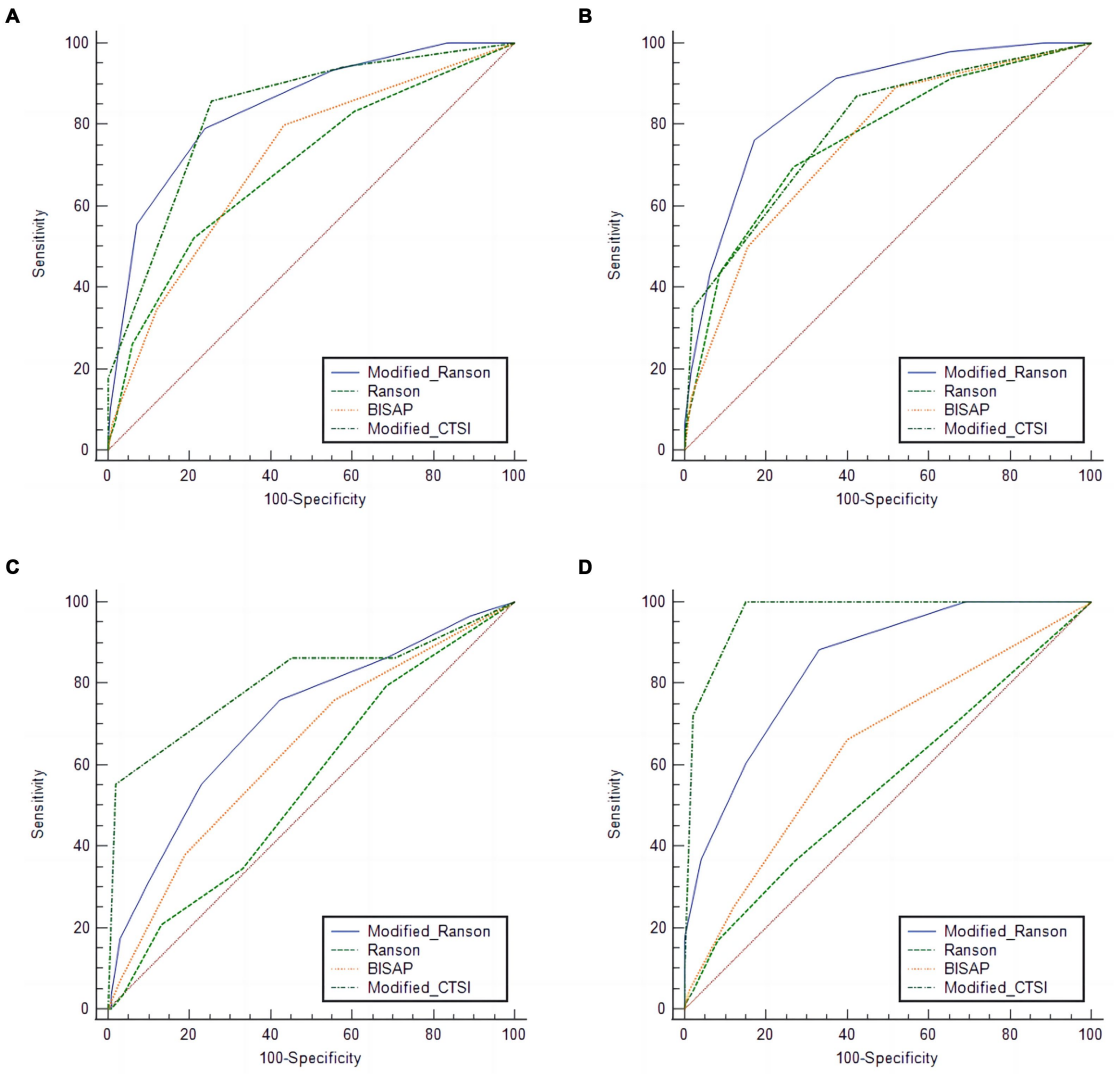
Receiver operating characteristic analysis of the modeling group (*n =* 304) for predicting pancreatitis severity **(A)**, organ failure **(B)**, pancreatic necrosis **(C)**, and **(D)**, pancreatic infection using four different scoring systems.

We compared the diagnostic accuracy of the four scoring systems for AP in prediction of four different outcomes in the modeling group: SAP, organ failure, pancreatic necrosis, and pancreatic infection (Figure 1; Table 3).

**Table 3:** Accuracy of the different scoring systems in predicting different outcomes in the modeling group (*n =* 304).

Outcome	Score system	AUC	95% CI	Sensitivity, %	Specificity, %	Cutoff value	Youden index	*p*-value
Severe pancreatitis	Modified Ranson	0.844	0.798, 0.883	78.99	76.22	≥3	0.5521	0.0001
	Ranson	0.695	0.640, 0.747	52.10	78.92	≥2	0.3102	0.0001
	BISAP	0.712	0.658, 0.762	79.83	56.76	≥1	0.3659	0.0001
	Modified CTSI	0.832	0.785, 0.872	85.72	74.59	≥2	0.6031	0.0001
Organ Failure	Modified Ranson	0.861	0.816, 0.897	76.09	82.95	≥4	0.5903	0.0001
	Ranson	0.765	0.713, 0.812	67.57	73.26	≥2	0.4282	0.0001
	BISAP	0.753	0.700, 0.800	89.13	48.06	≥1	0.3719	0.0001
	Modified CTSI	0.790	0.740, 0.835	86.96	57.75	≥2	0.4471	0.0001
Pancreatic necrosis	Modified Ranson	0.710	0.656, 0.761	75.86	57.82	≥3	0.3368	0.0001
	Ranson	0.551	0.494, 0.608	79.31	31.64	≥1	0.1095	0.3288
	BISAP	0.636	0.579, 0.690	75.86	44.36	≥1	0.2023	0.0094
	Modified CTSI	0.805	0.755, 0.848	58.17	98.18	≥3	0.5335	0.0001
Pancreatic infection	Modified Ranson	0.847	0.801, 0.885	88.24	67.00	≥2	0.5524	0.0001
	Ranson	0.552	0.495, 0.609	36.27	73.00	≥2	0.09275	0.1088
	BISAP	0.643	0.586, 0.696	66.18	60.00	≥1	0.2618	0.0001
	Modified-CTSI	0.970	0.944, 0.986	100	85.00	≥2	0.8500	0.0001

#### Severe-acute pancreatitis

For prediction of SAP, the modified Ranson score (cutoff: 3) had the greatest AUC (0.844) and the Ranson score had the lowest AUC (0.695). The modified Ranson score also had the second highest sensitivity (78.99%) and the highest specificity (76.22%). Comparison of the AUC values of each scoring system for prediction of SAP using the *Z*-test (Table 4) indicated the modified Ranson score had greater accuracy than the Ranson score and the BISAP score (both *p* < 0.05), but had similar accuracy as the modified CTSI score (*p* = 0.5850).

**Table 4:** Pairwise comparisons of the modified Ranson score with scores from the three other systems in the modeling group (*n =* 304).

Comparison group	Disease severity		Organ failure		Pancreatic necrosis		Pancreatic infection	
	*Z*	*p*-value	*Z*	*p*-value	*Z*	*p*-value	*Z*	*p*-value
Ranson	7.349	0.0001	3.459	0.0005	3.934	0.0001	15.253	0.0001
BISAP	5.196	0.0001	3.123	0.0018	1.418	0.1561	7.793	0.0001
Modified CTSI	0.585	0.5586	2.304	0.0212	3.496	0.0005	6.058	0.0001

#### Organ failure

For prediction of organ failure, the modified Ranson score (cutoff: 4) had the highest AUC (0.861), the third highest sensitivity (76.09%), and the highest specificity (82.95%). Comparison of the AUC values (Table 4) indicated the modified Ranson score had greater accuracy than the Ranson score, BISAP score, and modified CTSI score (all *p* < 0.05).

#### Pancreatic necrosis

For prediction of pancreatic necrosis, the modified Ranson score (cutoff: 3) had the second highest AUC (0.710), the second highest sensitivity (75.89%), and the second highest specificity (57.82%) Comparison of the AUC values (Table 4) indicated the modified Ranson score had greater accuracy than the Ranson score (*p* < 0.05), similar accuracy as the BISAP score, (*p* = 0.1561), and lower accuracy than the modified CTSI score (*p* < 0.05).

#### Pancreatic infection

For prediction of pancreatic infection, the modified Ranson score (cutoff: 2) had the second highest AUC (0.847), the second highest sensitivity (88.24%), and the third highest specificity (67.00%). Comparison of the AUC values indicated the modified Ranson score had greater accuracy than the Ranson score and the BISAP score (both *p* < 0.05), but lower accuracy than the modified CTSI score (*p* < 0.05).

### Roc analysis of the validation group using the four scoring systems

**Figure 2:**
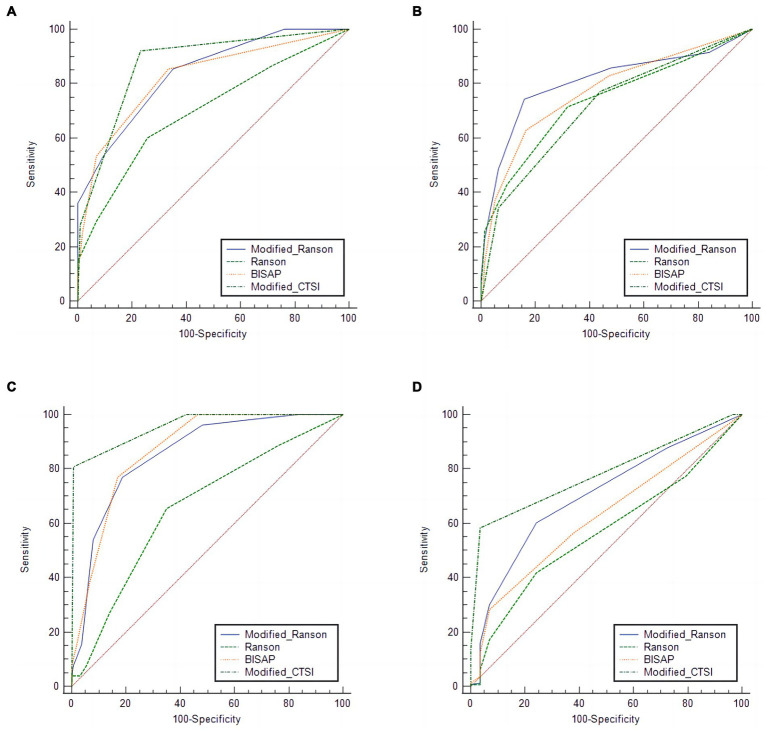
Receiver operating characteristic analysis of the verification group for predicting pancreatitis severity **(A)**, organ failure **(B)**, pancreatic necrosis **(C)**, and **(D)**, pancreatic infection using four different scoring systems.

We then used the same procedures to compare the diagnostic accuracy of the four scoring systems for prediction of the same four outcomes in the validation group (Figure 2; Table 5), and compared the different AUC values using the *Z*-test (Table 6).

**Table 5:** Accuracy of the different scoring systems in predicting different outcomes in the validation group.

Outcome	Score system	AUC	95%CI	Sensitivity, %	Specificity, %	Cutoff value	Youden index	*p*-value
Severe pancreatitis	Modified Ranson	0.839	0.780, 0.888	85.33	64.96	≥3	0.5029	0.0001
	Ranson	0.701	0.631, 0.765	60.00	74.36	≥2	0.3436	0.0001
	BISAP	0.825	0.763, 0.876	85.33	66.67	≥1	0.5200	0.0001
	Modified CTSI	0.873	0.818, 0.917	92.00	76.92	≥2	0.6892	0.0001
Organ failure	Modified Ranson	0.808	0.745, 0.861	74.29	84.08	≥4	0.5836	0.0001
	Ranson	0.738	0.670, 0.799	71.43	68.15	≥2	0.3958	0.0001
	BISAP	0.774	0.708, 0.831	62.86	83.44	≥2	0.4630	0.0001
	Modified CTSI	0.717	0.648, 0.780	77.14	56.05	≥2	0.3319	0.0001
Pancreatic necrosis	Modified Ranson	0.856	0.798, 0.902	76.92	81.33	≥4	0.5825	<0.000
	Ranson	0.657	0.585, 0.723	65.38	65.06	≥2	0.3044	0.0050
	BISAP	0.871	0.816, 0.915	76.92	83.13	≥2	0.6006	<0.0001
	Modified CTSI	0.956	0.917, 0.981	80.77	99.40	≥3	0.8017	<0.0001
Pancreatic infection	Modified Ranson	0.702	0.632, 0.766	60.12	75.86	≥3	0.3598	0.0001
	Ranson	0.567	0.493, 0.638	41.72	75.86	≥2	0.1758	0.1783
	BISAP	0.625	0.552, 0.693	28.22	93.10	≥2	0.2132	0.0073
	Modified CTSI	0.784	0.719, 0.840	58.28	96.55	≥2	0.5483	0.0001

**Table 6:** Pairwise comparisons of the modified Ranson score with scores from the three other systems in the verification group.

Comparison group	Disease severity		Organ failure		Pancreatic necrosis		Pancreatic infection	
	*Z*	*p*-value	*Z*	*p*-value	*Z*	*p*-value	*Z*	*p*-value
Ranson	6.032	0.0001	2.316	0.0208	5.184	0.0001	3.857	<0.0001
BISAP	0.454	0.6495	3.271	0.3918	0.554	0.5798	1.703	0.0886
Modified CTSI	1.108	0.2679	2.213	0.0192	3.76	0.0002	1.798	0.0721

For SAP, the modified Ranson score (cutoff: 3) had the second-highest AUC (0.839), was significantly more accurate than the Ranson score, and had similar accuracy as the BISAP score and modified CTSI score (*p* > 0.05). For organ failure, the modified Ranson score had the highest AUC (0.808), was significantly more accurate than the Ranson score and modified CTSI score (both *p* < 0.05), and had similar accuracy as the BISAP score (*p* = 0.6495). For pancreatic necrosis, the modified Ranson score had the third-highest AUC (0.856), was significantly more accurate than the Ranson score (*p* < 0.05), had similar accuracy as the BISAP score, and had lower accuracy than the modified CTSI score (*p* = 0.0002). For pancreatic infection, the modified Ranson score had the second-highest AUC (0.702), was significantly more accurate than the Ranson score (*p* < 0.0001) and had similar accuracy as the modified CTSI score (*p* = 0.0721) and the BISAP score (*p* = 0.5798).

## Discussion

AP is a systemic inflammatory disorder that is characterized by diverse signs and symptoms, rapid progression, and high mortality. This study compared 4 scoring systems—the Ranson score, BISAP score, modified CTSI, and a newly proposed modified Ranson score—for predicting severity of AP, organ failure, pancreatic necrosis, and pancreatic infection. The modified Ranson score was superior to all other scoring systems in predicting organ failure, and it had the highest specificity in predicting this outcome in the modeling group and the validation group. The AUC of our modified Ranson score for predicting the severity of AP was also highest in the modeling group, but was slightly inferior to the modified CTSI (but with no statistical difference) in the validation group. The modified Ranson score was second-best in predicting pancreatic infection and pancreatic necrosis, after the modified CTSI. This may be because the modified CTSI provides more direct information about pancreatic inflammation. The modified Ranson score includes serological indicators, which may lead to some bias in predicting pancreatic infection and pancreatic necrosis. The major results were that modified Ranson score provided more accurate prediction of AP severity, organ failure, pancreatic necrosis, and pancreatic infection than the original Ranson score.

The optimal cut-off of our modified Ranson score was 3 for predicting SAP in the modeling group and in the validation group. Thus, this study divided patients into two groups based on this cut-off value: a low-grade group (modified Ranson score <3) and a high-grade group (modified Ranson score ≥3). In terms of clinical outcomes (severity of AP, organ failure, pancreatic infection, and pancreatic necrosis) the high-grade group had worse outcomes (all *p* < 0.05). In addition, the high-grade group had a longer duration of hospitalization and a higher cost of hospitalization.

In the original 1974 study, the Ranson score had a sensitivity of 65% in predicting complications and mortality (9). The original purpose of the Ranson score was to identify alcoholic pancreatitis, but subsequent studies questioned its practicality due to individual differences among patients, and its poor specificity and poor sensitivity (13, 14). In particular, the Ranson score does not consider radiographic results, but it does include fluid sequestration, a measure that can be subjective and hard to collect and possibly lead to errors and reduced accuracy in predicting disease severity and patient prognosis. The newly proposed modified Ranson score includes radiographic but excludes fluid sequestration, and it provides a more comprehensive and objective assessment of AP severity.

The BISAP score requires fewer variables that the Ranson score, making it easier to calculate. However, the BISAP score requires assessment of mental status, a subjective measure, and measurement of baseline mental status, making its use problematic for patients with a long course of disease. Fortunately, all the variables included in the newly proposed score are objective routine clinical indicators, thus reducing the subjective bias of evaluators, which could be used between different centers.

The CTSI has relatively good sensitivity and specificity for predicting the severity of AP and patient prognosis, but its accuracy is lower for prediction of organ failure and extra-pancreatic complications (15, 16). To address these shortcomings, researchers developed the modified CTSI, which provides better assessment of pancreatic necrosis and extra-pancreatic complications (17). The modified CTSI has higher sensitivity but lower specificity than the CTSI in stratifying disease severity (18). However, the modified CTSI score also has limitations. First peripancreatic necrosis and pseudocyst formation are typically rare in patients with early-stage AP. Second, the evaluation needs to be conducted 2 to 3 days after admission, possibly leading to delays in treatment (19). Doing a CT scan within 48 h because the time node of the modified Ranson score in this study was 48 h, and which was consistent with the time node of define about severity of AP. Therefore, CTSI scores within 48 h of onset were compared with new score in this study which might limit its applications in clinical practice. The modified Ranson score combines biochemical data and radiological data, evaluation is performed in 48 h, and it provides full assessment of patients whose disease severity may be difficult to determine. Therefore, the newly proposed modified Ranson score provides reliable, comprehensive, and objective assessment of the severity of AP.

At present, in addition to the above traditional score, many scholars have applied artificial intelligence (AI) models to predict the severity of acute pancreatitis. Balazs et al. (20) developed the EASY prediction score using machine learning models. The EASY prediction score is a practical tool for identifying patients at high risk for severe AP within hours of hospital admission. The AI model developed by Keogan et al. (21) was compared to the CTSI and Ranson scores, both of which were found inferior in terms of predicting the severity of AP. Despite the tremendous efforts and scientific results, much of this knowledge has not been applied in everyday clinical practice (22). In order to bring these complex models to the bedside, they need to be implemented as easy to use and broadly accessible tools (23). In the future, it may be possible to combine modified Ranson score with AI to develop a model for predicting the severity and prognosis of acute pancreatitis.

In the 40 years since the Ranson score system was first introduced, various scoring systems have been suggested; however, to date, none can accurately predict severity at an early stage, are non-invasive, or easy to use in patients (24). A number of studies have compared existing Scoring Systems in identification patients at risk for SAP (24–28). In addition, there are many studies aimed at exploring simple, effective and accurate prediction models. Lei Wang developed a new Chinese simple scoring system (CSSS) that comprises only 6 variables were collected within 48 h of admission (25). In this study, the new scoring system was the most accurate in predicting disease severity according to the AUC, followed by APACHE II, Ranson score, MCTSI, and BISAP. In particular, studies have compared the predictive effectiveness of scoring systems in specific populations (26, 28). A study compared several scoring systems in predicting SAP of patients with hypertriglyceridemia-induced acute pancreatitis (HTG-AP) which showed that all score systems had medium performance in predicting SAP and pancreatic necrosis in HTG-AP (26). Others have studied AP in the elderly (28). It figured out BISAP is the most appropriate scoring system for the prediction of severity. Ranson and APACHE II for elderly patients are not as useful as they are for younger patients. To sum up, although several scoring systems have been developed, each system has its specific applications and limitations. It is of clinical significance to develop a new and effective scoring system to predict the severity and mortality of SAP. And our study constructs a new AP scoring system based on the analysis of patients with SAP and it has perfect predictive effectiveness.

In conclusion, the newly proposed modified Ranson score is more comprehensive and objective. Compared to three existing scoring systems, it is accurate in predicting disease severity (MSAP and SAP), especially for predicting organ failure which plays important roles in early evaluation, progress analysis, treatment plan adjustment, and prognosis judgment of AP. There was a study that showed APACHE II can be a useful tool in predicting which patients are likely to develop severe disease early in the course of their illness (27). But the APACHE II score is not an evaluation for a specific disease. Instead, the APACHE II score is an indicator used to classify patients who need to be treated in an intensive care unit (ICU). In this study, APACHE II score was not included because most of the patients included in this study were mild and the score had many indicators, which were difficult to collect completely and could not be scored. A limitation of this study is that it was a single-center, retrospective study with small sample size, factors that may lead to bias. Thus, our conclusions regarding the accuracy of the modified Ranson score need to be verified by large and multi-center studies. A second limitation is that some of the scoring systems we evaluated had parameters that were somewhat subjective, and this could also lead to some bias.

## Conclusion

The modified Ranson score described here provided better overall diagnostic performance in predicting severe AP, organ failure, pancreatic necrosis, and pancreatic infection than the original Ranson score. In addition, our modified Ranson score provided more accurate predictions organ failure than the three other AP scoring systems.

## Data availability statement

The raw data supporting the conclusions of this article will be made available by the authors, without undue reservation.

## Ethics statement

This study was approved by the Ethical Review Committee of the Second Affiliated Hospital of Guangxi Medical University [Approval Number: 2020-KY (0136)].

## Author contributions

XL and JW conceived and coordinated the study and wrote the manuscript. QW, PP, GL, CL, and HY were responsible for data collection, and data analysis, respectively. JH and MQ reviewed and revised the manuscript. All authors checked the results and approved the final manuscript. All authors contributed to the article and approved the submitted version.

## Funding

This study was funded by the Scientific Research Project of Guangxi Health and Family Planning Commission (Z20210625), Guangxi Traditional Chinese Medicine Research Project (GXZYA20220227) and Science Foundation Project of the Second Affiliated Hospital of Guangxi Medical University (EFYKY2020007).

## Conflict of interest

The authors declare that the research was conducted in the absence of any commercial or financial relationships that could be construed as a potential conflict of interest.

## Publisher’s note

All claims expressed in this article are solely those of the authors and do not necessarily represent those of their affiliated organizations, or those of the publisher, the editors and the reviewers. Any product that may be evaluated in this article, or claim that may be made by its manufacturer, is not guaranteed or endorsed by the publisher.

## Abbreviations

AP: Acute pancreatitis
SAP: Severe acute pancreatitis
APACHE-II: Acute Physiology and Chronic Health Evaluation II
BISAP: Bedside Index of Severity in Acute Pancreatitis
CTSI: Computed Tomography Severity Index
